# A Random shRNA-Encoding Library for Phenotypic Selection and Hit-Optimization

**DOI:** 10.1371/journal.pone.0003171

**Published:** 2008-09-09

**Authors:** Yongping Wang, Yun E. Wang, M. Grazia Cotticelli, Robert B. Wilson

**Affiliations:** 1 Department of Pathology and Laboratory Medicine, University of Pennsylvania, Philadelphia, Pennsylvania, United States of America; 2 School of Arts and Sciences, University of Pennsylvania, Philadelphia, Pennsylvania, United States of America; The Research Institute for Children at Children's Hospital New Orleans, United States of America

## Abstract

RNA interference (RNAi) is a mechanism for inhibiting gene expression through the action of small, non-coding RNAs. Most existing RNAi libraries target single genes through canonical pathways. Endogenous microRNAs (miRNAs), however, often target multiple genes and can act through non-canonical pathways, including pathways that activate gene expression. To interrogate all possible functions, we designed, synthesized, and validated the first shRNA-encoding library that is completely random at the nucleotide level. Screening in an IL3-dependent cell line, FL5.12, yielded shRNA-encoding sequences that double cell survival upon IL3 withdrawal. Using random mutagenesis and re-screening under more stringent IL3-starvation conditions, we hit-optimized one of the sequences; a specific nucleotide change and the creation of a mismatch between the two halves of the stem both contributed to the improved potency. Our library allows unbiased selection and optimization of shRNA-encoding sequences that confer phenotypes of interest, and could be used for the development of therapeutics and tools in many fields of biology.

## Introduction

Small, non-coding RNAs can inhibit gene expression through interaction with mRNAs in a process called RNA interference (RNAi) [1]. In the canonical pathway, hairpin-loop microRNAs (miRNAs) transcribed from the genome are processed by the ribonucleases Drosha and Dicer into ∼22-nucleotide (nt) small-interfering RNAs (siRNAs). The RNA-Induced Silencing Complex, RISC, uses the siRNAs to cleave and/or inhibit the translation of complementary mRNAs in a sequence-specific manner. Transfection of cells with chemically synthesized siRNAs can produce a transient silencing of appropriately targeted mRNAs. Expression of short-hairpin-loop RNAs (shRNAs), which are modeled on miRNAs, can be used for sustained RNAi.

Many endogenous miRNAs target short sequences in the 3′ untranslated regions (UTRs) of a large number of mRNAs. Over-expression of the miRNAs miR-1 and miR-124, for example, shifted cellular expression profiles to those of muscle and brain, respectively [2], with apparent targeting to sequences of only seven and six nucleotides, respectively, in the 3′ UTRs of the mRNAs. Vertebrate miRNA-target interactions are mediated primarily by “seed” matches of six nucleotides (miRNA nucleotides 2–7) supplemented with either a U at position 1 or a match at position 8 [3]. Hence sequence matches of fewer than eight nucleotides are apparently sufficient to exert phenotypic effects, likely through translational repression [4]–[6]. If the complementarity extends beyond the 10^th^ nucleotide of the miRNA, target mRNA cleavage occurs [7], [8].

Our understanding of the full range of effects of miRNAs, shRNAs, and siRNAs is far from complete, and non-canonical functions are becoming apparent. For example, recent findings indicate that small RNA species can *activate* gene expression. Promoter-targeted siRNAs activated expression of the progesterone receptor in a sequence-specific manner [9]. Promoter-targeted siRNAs also activated the genes encoding E-cadherin, p21, and vascular endothelial growth factor [10]; the seed sequence and Dicer were required, and the activation was sequence specific. Three independent miRNAs targeted to the 3′ UTRs of three different mRNAs repressed translation in proliferating cells but activated translation in cell-cycle-arrested cells; the translational activation required both FXR1 and AGO2, which exhibited a miRNA-dependent association with the mRNAs [11].

RNAi is now commonly used as a tool to knock down the expression of specific genes through rational-design algorithms targeting specific sequences in mRNAs [12]. RNAi libraries based on canonical pathways have been developed for screening purposes. Most of these libraries encode shRNAs and target single genes with each construct. Some of these libraries focus on particular pathways or gene-sets of interest [13], [14], while others are broad-based [15], [16]. Second-generation libraries have been constructed in miRNA contexts for improved RNAi effects [17]. In part to decrease costs associated with generating thousands of individual constructs by rational design, several groups have used enzyme-based approaches to construct RNAi libraries from either cDNA or genomic DNA fragments [18]–[21]. These RNAi libraries are useful for identifying single genes of biologic interest, or genes that encode potential targets for conventional drug development. However, for identifying shRNAs or siRNAs to be used in and of themselves as therapeutics or biologic tools, the most effective sequences may target more than one gene and/or may act through non-canonical mechanisms. To identify such sequences, libraries that are random at the nucleotide level, and therefore unbiased with respect to mechanism of action, are preferable.

One group created a library that uses two opposed promoters to transcribe linear RNAs from the same 19-base-pair random sequence simultaneously [22], which may limit transcriptional efficiency and lead to effects based on antisense. Even assuming siRNA effects, several limitations remain: 1) siRNAs are less potent than shRNAs [23], [24]; in the context of screening random libraries, potency is critical since the initial effects are expected to be weak. 2) The design of siRNA-encoding libraries precludes mismatches in the RNA duplex, a factor contributing to the potency of endogenous miRNAs. 3) shRNAs or siRNAs with 27–29 bp stems are far more potent inducers of RNAi than constructs with 19–21 bp stems [25], [26]; however, siRNAs >23 bp in length are more likely to induce, non-specific interferon responses [27].

To overcome the limitations of single-gene-targeting shRNA libraries and the aforementioned random, siRNA-encoding library, we set out to design, synthesize, and test a random, shRNA-encoding library. The primary goals of the design were to allow the expression of random shRNAs with stems of optimal length (29-nt [25]); to allow the straightforward retrieval of “hit” sequences by PCR and cloning back into the vector; and to allow mismatches between the two halves of the stem after random mutagenesis for hit-optimization. The synthetic challenges included, 1) creating the reverse complement of a 29-nt random sequence in the same strand of DNA, 2) creating a non-complementary loop between them, 3) synthesizing a double-stranded version of this DNA, and 4) cloning the double-stranded version into an appropriate vector for robust expression, with a polymerase termination sequence at the 3′ end and a poly-pyrimidine tract upstream of the transcription start site. Herein we describe how we solved these technical challenges and used a functional approach to identify hit sequences that protect an IL3-dependent cell line from IL3 withdrawal, and how we hit-optimized by random mutagenesis and re-screening.

## Results

### Library synthesis and characterization

**Figure 1:**
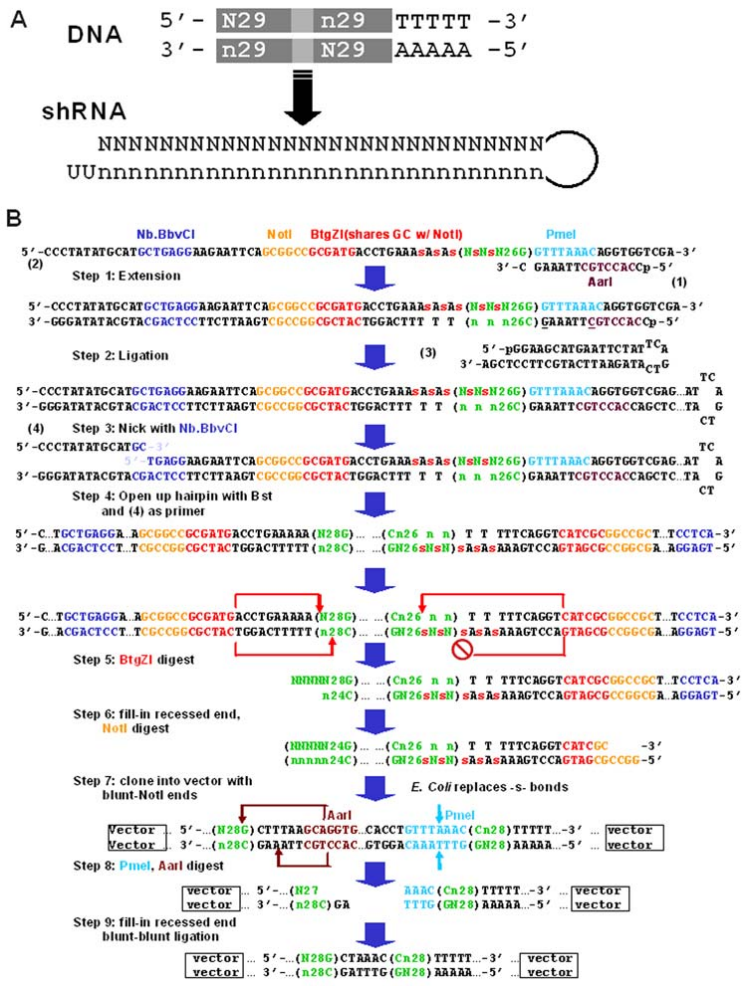
Synthesis of the random shRNA library. (A) Schematic diagram of the DNA cassette encoding the shRNA transcript, where N and n indicate complementary bases. (B) The steps of library synthesis (see Materials and Methods for details). Step 1: Extension using oligo (1) as primer and oligo (2) as template. The underlined mismatches create an intact *Aar*I site and mutated *Pme*I site in the oligo (2). The letter “s” in oligo (2) indicates a phosphorothioate linkage. Step 2: Ligation to short hairpin-loop (3). Step 3: Digestion of one strand by nicking enzyme Nb.BbvCI. Step 4: Opening of the large hairpin-loop by extension using strand-displacing polymerase *Bst*, and additional oligo (4) as primer. Step 5: Digestion with *Btg*ZI, which cuts downstream of its recognition site at one end, but not the other, because of the phosphorothioate linkages. Step 6: Fill-in of the *Btg*ZI-cut end and digestion by *Not*I of the other end. Step 7: Cloning of the blunt-*Not*I cassette into an shRNA-expression vector, and transformation into *E. coli*, which replace the phosphorothioate linkages with normal phosphodiester linkages. Step 8: Asymmetric digestion with *Pme*I and *Aar*I, because of the designed mismatches between the first primer (1) and the original template oligo (2). Step 9: Fill-in of the *Aar*I end and unimolecular blunt-blunt ligation, resulting in an asymmetric loop sequence.

The synthesis of our random, shRNA-encoding library is described in detail in the Materials and Methods section and shown in Figure 1. Figure 1A shows the essential elements of the double-stranded DNA cassette encoding the random shRNAs. The sense strand comprises a random 29-nt sequence (“N29”), a non-complementary loop sequence, the reverse complement (“n29”) of the random 29-nt sequence, and a polymerase III termination sequence. The rationale for a 29-nt stem comes from the work of Siolas *et al.*, who showed that shRNA constructs with 29-nt stems and 2-nt 3′ overhangs were far more potent inducers of RNAi than equivalents with 19-nt stems [25]. The construction of our library requires a fixed base at the 29^th^ position, which we arbitrarily made a Guanine initially (Figure 1B). This “N28+G” library represents one-fourth of a library encoding shRNAs with random, 29-nt stems; the other three-fourths could be synthesized by altering the Guanine at the 29^th^ position to each of the other three bases in turn. Because Dicer removes the 29^th^ position in canonical RNAi processing, we opted to proceed with the construction and testing of the “N28+G” library. Because the initial library was prepped from ∼300,000 *E. coli* colonies (to ensure adequate coverage of 4^8^ or 65,536 possible seed sequences), we refer to it herein as the “300K library.” To rule out significant sequence bias introduced by any of the steps during synthesis, we sequenced 40 individual clones, either from the final library or from the intermediate prior to the creation of the non-complementary loop. The AT composition was 52.8% (14.78 of 28) and the GC composition was 47.2% (13.22 of 28) (Figure S1B). The DNA cassette was initially designed for cloning into the retroviral expression vector pSuper (OligoEngine). We later transferred the library to pSiren (Clontech), which provided better expression of green fluorescent protein (GFP). The preparation of both vectors, and the *en bloc* move of the library from pSuper to pSiren, are described in Figure S2.

### Selection for shRNAs that inhibit apoptosis

The murine pro-B cell line FL5.12 is IL3 dependent; ∼100% of the cells die by apoptosis after IL3 withdrawal for 2–3 days and >90% of cells can be rescued by Bcl-xL [28]. We infected ∼6 million FL5.12 cells with the library to ∼30% GFP positivity. The rationale for ∼30% infectivity was to ensure the delivery ∼1 shRNA construct per cell, thereby maximizing the chances of identifying effective shRNA sequences with even weak effects. (Most of the shRNAs encoded by the library are presumably inactive, but could compete with effective sequences for loading onto RISC.)

**Figure 2:**
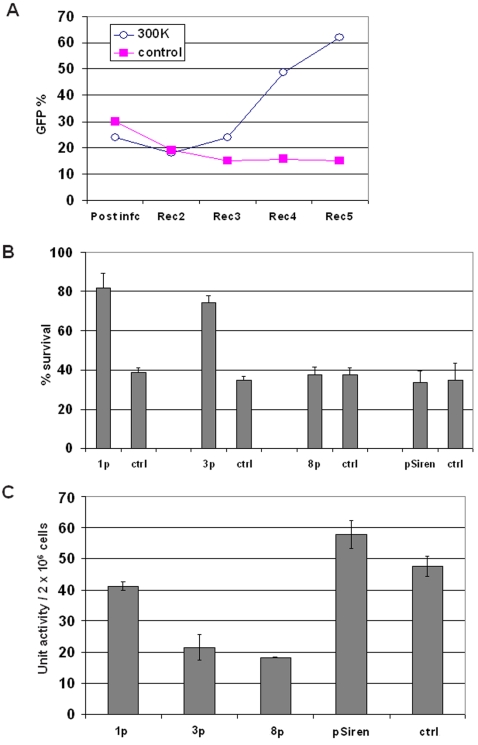
Selection for shRNAs that protect FL5.12 cells from IL3 withdrawal. (A) GFP percentages of FL5.12 cells infected with a random-shRNA-expressing control or the 300K library and subjected to repeated IL3 starvation/recovery (3day/3day) cycling. The GFP percentages are shown post infection (infc) and after each recovery (Rec). (B) Survival percentages (percentages of GFP+/PI− cells) shown are relative to the beginning of IL3 starvation. Three shRNA constructs – 1p, 3p, and 8p – were tested against a random shRNA control or empty vector (pSiren) in FL5.12 cells starved for 14 hours. By Student's t-test, we found *p*<0.001 for 1p and 3p vs. controls. Data are means +/− standard deviations (SD). 8p offered similar ∼2× protection relative to control when tested several days later (Figure S3). (C) Caspase 3 activities of cells infected with 1p, 3p, 8p compared to control shRNA and pSiren at 12 hours of IL3 starvation. By Student's t-test, we found *p* = 0.031 for 1p vs. control, and *p*<0.001 for 3p and 8p vs. control. Data are means +/− SD.

After 3 days in IL3-negative medium, surviving cells were transferred back to regular growth medium with 0.3 ng/ml IL3 for 3 days. To enrich for true positives, we withdrew IL3 again and the process was repeated. (The mathematics of enrichment scenarios are shown in Table S1) After four rounds of IL3 withdrawal and recovery, the percentage of GFP-positive cells in the library-infected wells (but not in the control-infected wells) rose to 60%, suggesting the presence of hit sequences that conferred a relative survival advantage (Figure 2A). (We consistently observed a post-infection decline in the percentage of GFP-positive cells suggesting that a subset of shRNAs, GFP, and/or the infection treatment itself were detrimental to the cells). We isolated genomic DNA, amplified by PCR using pSiren-specific primers flanking the hairpin-loop insert, and cloned back into pSiren.

Of 10 shRNA-encoding sequences we sequenced, two were the same (clones “1p” and “7p”), suggesting selective enrichment. We infected FL5.12 cells with three of the putative hit sequences (1p, 3p, and 8p) in six independent infections for each clone and withdrew IL3 for 14 hours (to allow weak effects to be discerned). We analyzed the cells by flow cytometry, identifying infected cells by GFP fluorescence and dead cells by propidium iodide (PI) staining. The fractions of GFP-positive (infected), PI-negative (live) cells relative to the start of the experiment (just prior to IL3 withdrawal) are shown in Figure 2B. Clones 1p and 3p conferred a statistically significant improvement in survival relative to cells infected with a random control shRNA (“ctrl”) or with vector alone (pSiren) (*p*<0.0001 in each case by Student's t-test). Clone 8p did not confer protection initially (Figure 2B); however, after several days of culturing in IL3, 8p conferred protection from IL3 withdrawal similar to that of 1p and 3p (Figure S3, note the longer starvation time of 22 hours in this assay). All three clones were tested for effects on growth rate and none were seen (Figure S4). The sequences of 1p, 3p, and 8p, including the CTAAAC loop, are:

Clone 1p 5′-GGGTAGCTACATTTGCATATGTGGATATG CTAAAC CATATCCACATATGCAAATGTAGCTACCC-3′

Clone 3p 5′-GTGGATCAGTGTGTTATAGCTCGGGCAGG CTAAAC CCTGCCCGAGCTATAACACACTGATCCAC-3′

Clone 8p 5′-GGCGGCGGCAAGGAAGGCATTGAGGACTG CTAAAC CAGTCCTCAATGCCTTCCTTGCCGCCGCC-3′

**Figure 3:**
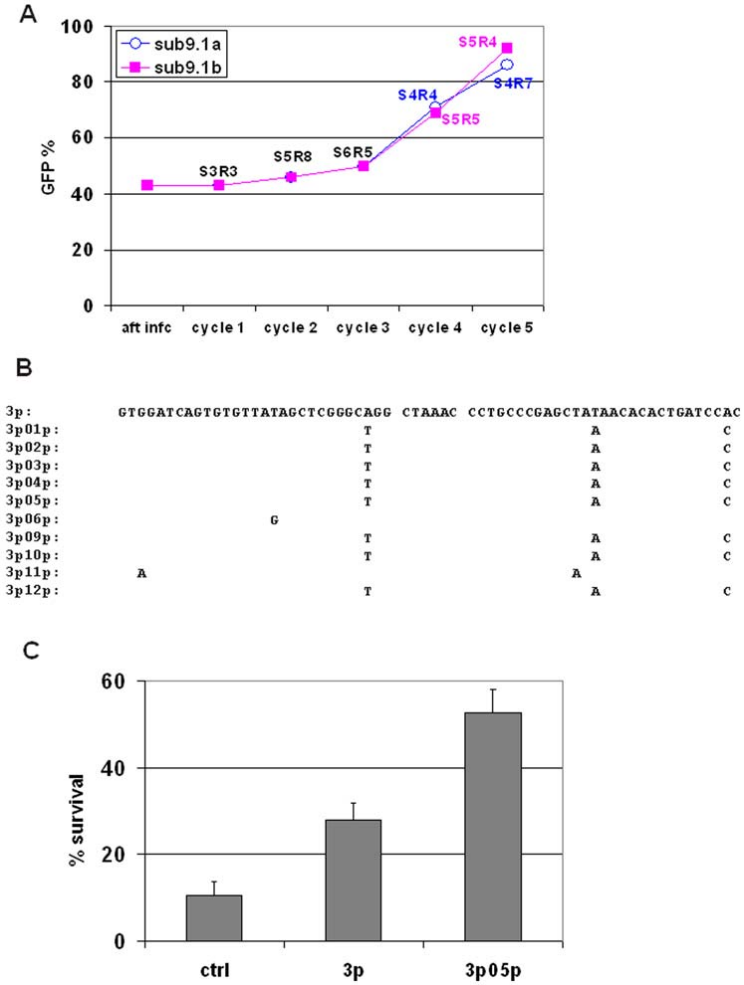
Hit-optimization of 3p. (A) GFP percentages of FL5.12 cells infected with sublibrary 9.1, which was synthesized by random mutagenesis of the sequence of 3p. FL5.12 cells were subject to extended IL3 starvation/recovery cycles; “SxRy” represents Starvation for x days and Recovery for y days. After the third cycle, the cells were subjected to either four- or five-day starvations, and the clones recovered were called sub-library 9.1a and 9.1b, respectively. (B) Sequences of ten clones from sublibrary 9.1b retrieved from FL5.12 cells surviving extended IL3 withdrawal. (C) Survival percentages (percentages of GFP+/PI− cells) are shown for cells infected with 3p05p, 3p, and a random control shRNA (ctrl), after 22 hours of IL3 starvation. By Student's t-test, we found *p* = 0.014 for 3p05p vs. 3p, and *p*<0.01 for 3p vs. control. Data are means +/− SD.

**Figure 4:**
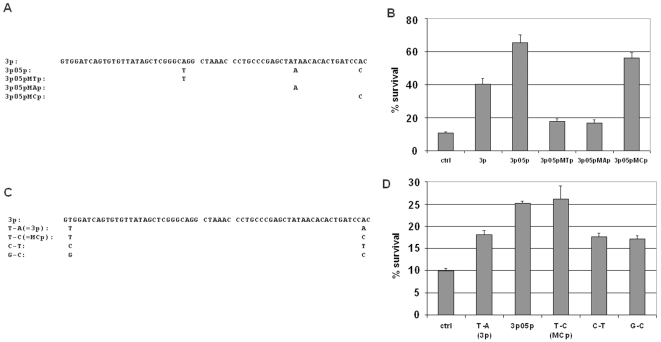
Sequence variation outside the “seed” region as well as mismatches are important for protection from apoptosis. (A) Sequences of three single-mutation variants of 3p based on the sequence of 3p05p. (B) The A-to-C mutation accounts for most of the improved activity relative to 3p. Survival percentages (percentages of GFP+/PI− cells) are shown for cells infected with 3p05p, 3p, a random control shRNA (ctrl), and three single-mutation variants of 3p based on the sequence of 3p05p, after 20 hours of IL3 starvation. Among the variants, only 3p05pMCp is more protective than 3p (p<0.0001); the other two variants (MTp, and MAp) are less protective than 3p (p<0.0001 for both). Data are means +/− SD. (C) Sequences of two additional variant clones: C-T and G-C. (D) Increased activity of MCp (T-C) over 3p (T-A) (p = 0.002) was abolished by either variant C-T (nucleotide “C” changed, but mismatch retained) or G-C (“C” retained, but mismatch eliminated) (p<0.001 for both). Data are means +/− SD.

**Figure 5:**
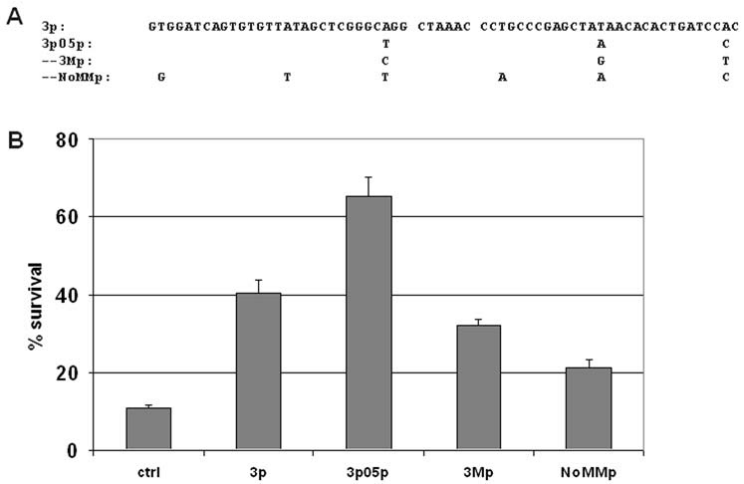
Variants of 3p05p abolish its activity and suggest the importance of mismatches and sequence specificity. (A) Three nucleotides were changed in 3p to form 3p05p (T-A-C), thus introducing mismatches between the two halves of the stem. Clone 3Mp was designed to maintain the three mismatches, but by changing T-A-C to C-G-T. Clone NoMMp was designed to keep the T-A-C, but to eliminate the mismatches by altering the corresponding nucleotides on the other half of the stem to G-T-A. (B) FL5.12 cells were transduced with the shRNA clones shown, and subjected to IL3 withdrawal for 20 hours. Both variants of 3p05p lost their protective activities, to a level even lower than that of 3p. (p<0.001 for any pair-wise comparisons in the figure) Data are means +/− SD.

Longer IL3 withdrawal times were associated with lower survival percentages, but the hit shRNAs consistently conferred approximately double the survival relative to a control random shRNA or empty vector (see figures 2B, 3C, 4B, 4D, 5B, and S3, S5, which show survival percentages assayed at 14–22 hours). The protective effects of 1p, 3p, and 8p upon IL3 withdrawal were largely intact after continuous culture for four months in IL3-containing medium, and a random sample of 10 individual clones from the 300K library failed to protect FL5.12 cells from IL3 withdrawal (Figures S5 and S6). Because FL5.12 cell death after IL3 withdrawal is apoptotic, we tested whether the protective effects of the hit shRNAs were associated with a decrease in the activity of caspase 3. Caspase 3 activity was markedly decreased in cells infected with 3p and 8p, but less so in cells infected with 1p (Figure 2C, *p* = 0.031 for 1p vs control, and *p*<0.001 for 3p and 8p vs control). Using BLAST, and readily available miRNA target predicting software (miRGen, miRanda, TargetScan, and MiRScan), we were unable to identify convincing targets for 1p, 3p, or 8p.

### Hit-optimization

We created random variants of the 3p shRNA sequence using an oligo synthesizer and spiking each phosphoramidite (A, C, G, T) bottle with the other three (“spiked oligo synthesis”) [29] at a low concentration designed to generate ∼1.5 mutations per construct after annealing the two complementary DNA strands. These mutations could occur anywhere in the cloning cassette, including the stem, the loop, and sequences outside the stem-loop. We cloned these 3p variants into pSiren to create a sublibrary. Because approximately 3000 clones are needed to cover 1.5 mutations on average, we harvested ∼10,000 E. coli colonies for the sublibrary, which we named sublibrary 9.1.

We introduced sublibrary 9.1, 3p, and a random control shRNA into FL5.12 cells by retroviral infection, and subjected the cells to cycles of IL3 “starvation and recovery” just as with the original library except that we increased the stringency of selection by extending the length of the IL3 starvations. The first starvation-recovery cycle comprised three days of IL3 starvation and three days of recovery (S3R3); the second comprised five days of IL3 starvation and eight days of recovery (S5R8) (Figure 3A). Neither the cells infected with a random control shRNA nor the cells infected with 3p survived the second cycle. After the third cycle, the cells were subjected to either four- or five-day starvations, and the clones recovered were called sub-library 9.1a and 9.1b, respectively. The percentage of GFP-positive cells rose sharply to 70% after the fourth cycle, and to >85% after the fifth cycle (Figure 3A). Of 10 shRNA-encoding sequences we retrieved from sublibrary 9.1b after the fifth cycle, eight were identical, suggesting selective enrichment (Figure 3B). We tested whether one of the eight identical clones (“3p05p”) was more efficacious than 3p at protecting FL5.12 cells from IL3 withdrawal, except that we determined percent survival after 22 hours of IL3 withdrawal rather than after 14 hours. 3p05p significantly improved survival relative to 3p, and 3p was still approximately twice as protective as control (Figure 3C, *p* = 0.014 for 3p05p vs 3p, and *p*<0.01 for 3p vs control).

There are three nucleotide differences between 3p05p and 3p, which introduced both sequence changes and mismatches between the two halves of the stem. We therefore synthesized variants of 3p05p, each of which has only one of the three nucleotide changes (Figure 4A), and quantified their activities in protecting FL5.12 cells from IL3 withdrawal. The variant with the A-to-C change, MCp, was more protective than 3p (*p*<0.0001), and was nearly as protective as 3p05p, whereas the activities of the other two variants (MTp and MAp) were less than that of 3p (Figure 4B, *p*<0.0001 for each). These results suggest that the A-to-C change accounts for most of the improved effect of 3p05p, and that the effect of one or both of the other two nucleotide changes depends on sequence context. The beneficial C of MCp (Figure 4A) creates a mismatch with the other half of the (3p) stem.

To test whether the mismatch per se, or the presence of the specific nucleotide C, contributed to the improved activity, we synthesized and tested two additional variants. In one variant (clone “G-C”), we maintained the nucleotide C of MCp, but eliminated the mismatch by placing a complementary G in the other half of the stem. In the other variant (clone “C-T”), we changed the critical nucleotide C to a T but maintained the same mismatch as that found in MCp by placing a non-complementary C in the other half of the stem (Figure 4C). The improved activity of MCp (T-C) relative to 3p (T-A) was abolished by the alteration in clone G-C, as well as by the alteration in clone C-T (Figure 4D, *p*<0.001 for both), suggesting that both the mismatch *and* the presence of the specific nucleotide C contributed to the improved activity.

We also synthesized and tested variants of 3p05p (Figure 5A) in which we eliminated the three mismatches by altering the corresponding nucleotides on the opposite half of the stem (NoMMp), or in which we retained the three mismatches but changed each of the two new pyrimidines in 3p05p to a different pyrimidine and the new purine to a different purine (3Mp). The activity of 3p05p was abolished in both variants, though more so in the NoMMp clone (Figure 5B), again suggesting the importance of mismatches (though we cannot rule out that the complementary base changes in the other half of the stem in NoMMp affected processing by Dicer or loading onto RISC).

## Discussion

A potential limitation of the random shRNA approach is that there are approximately 18 trillion (4^22^) random 22-nt sequences that could be loaded onto RISC. Selection assays using 18 trillion cells are infeasible, and multiple sequences introduced into individual cells would compete with each other for loading onto RISC. Hence, only a fraction of the total possible 22-mer sequences can be interrogated. What makes the random approach viable, at least for the canonical RNAi pathway, is the importance of the seed match in target interactions. Infection and analysis of several million cells is straightforward, yet interrogation of only ∼65,500 random shRNAs ensures a U at position 1 *and* a match at positions 2–7 *and* a match at position 8. The critical factors in the random approach are to ensure that, at a minimum, all possible seed matches are covered, and to ensure that the potency of the constructs is maximized; shRNAs that match only seed sequences may produce only weak effects, but can be hit-optimized by random mutagenesis and re-screening, as evidenced by the data herein.

The primary advantage of the random shRNA approach is that it is unbiased with respect to mechanism(s) of action, of which our understanding remains incomplete. It is of interest to note that in the mutagenesis study (Figure 4), the critical “C” mutation occurred at the 3′ end of the shRNA, outside the “seed region,” and the “T” mutation, which made MTp even less active than 3p, occurred outside the canonical 22-mer sequence of a mature miRNA. Taken together, these results not only point to the importance of both sequence specificity and mismatches between stems in the activity of shRNAs, they also illustrate the power of our library design and mutagenesis approach to select for the most active shRNAs in an agnostic manner. For shRNA-based therapeutics or biologic tools, the most effective sequences may target more than one gene and/or may act through non-canonical mechanisms. After infection or transfection with a random shRNA-encoding library, cells with a desired phenotype can be selected functionally (by survival or flow-sorting, for example), and effective sequences can be retrieved by PCR. In effect, we allow the cells to determine which sequences are the most effective, and the least toxic, without prior assumptions.

For functional selection, our random, shRNA-encoding library has specific advantages over the random, siRNA-encoding library that has been described [22]. In addition to the advantages described in the introduction, we have demonstrated herein the importance of mismatches in contributing to the potency of RNA duplexes; the design of the siRNA library precludes mismatches because the siRNAs are encoded by opposing DNA strands and thus are eliminated in *E. coli* during cloning. The combined benefits of shRNAs over siRNAs are critical for random-library screens, in which initial hits are expected to be weak. The shRNAs encoded by our library more closely resemble endogenous miRNAs, and, as such, maximize chances for success.

Our unbiased, functional approach complements existing RNAi libraries, which target single genes. Single-gene targeting is well suited for traditional drug development, since the encoded protein might be targeted using small molecules, which involves an additional screening process, followed by synthetic chemistry and re-screening for hit-optimization. Our approach is designed to identify sequences that can be used as therapeutics or biologic tools in and of themselves. In this regard, hit-optimization through random mutagenesis is significantly easier than the synthetic chemistry required to modify small molecules in traditional hit optimization. Although initial hits from single-gene-targeting RNAi libraries may be more potent than initial hits from our library, the number of target genes for the most potent miRNAs is thought to be in the hundreds, suggesting that shRNA potency for inducing a specific phenotype may derive from altering the expression of a large number of genes partially, instead of altering the expression of a single gene considerably. A possible reason for this increased potency is the tendency for cells to compensate homeostatically for a significant change in the expression of a single gene. Particularly in the context of tissue identity and differentiation, the miRNA paradigm [2] indicates that targeting multiple genes is often more effective to achieve a particular phenotype. In addition, our library allows for the exploration of non-canonical mechanisms not addressed by existing libraries.

It is unsurprising that we were unable to identify convincing targets for our shRNAs using target-identification algorithms such as BLAST, miRGen, miRanda, TargetScan, and MiRScan. The sequence space we were interrogating comprised an entire genome, and the shRNA sequences we identified were selected from a random library, are unlikely to exist in nature, and may act by non-canonical mechanisms. In addition, given the miRNA paradigm, the shRNA sequences we identified are likely to have many targets and are likely to have mismatches with some or all of their canonical targets. As our knowledge increases – of the mechanisms of action of shRNAs and of the sequence rules governing these mechanisms of action – it may be possible to identify critical targets of shRNAs selected functionally from our library. Microarray analyses of cells expressing these shRNAs might help by narrowing the sequence space to be interrogated using improved target-identification algorithms to the set of genes whose expression changes significantly, though these changes may derive from direct or indirect actions of the shRNAs. In many cases, identification of the most critical targets may be problematic. However, our primary goal in developing a random, shRNA-encoding library was to identify potential shRNAs to be used as therapeutics and biologic tools, rather than potential targets for conventional drug development. For applications in which transient or reversible effects are need, shRNA sequences identified and optimized by our approach could be used as siRNAs.

For shRNA and siRNA therapeutics, issues of delivery to specific tissues remain [30], but are increasingly being solved. For example, Kumar *et al.* used a 29-residue peptide from the rabies virus glycoprotein envelope to deliver siRNAs from the bloodstream specifically to neurons, thereby protecting mice against subsequent Japanese encephalitis virus infection [31]. Several companies are already involved in clinical trials of RNAi therapeutics [32], including Acuity (age-related macular degeneration, diabetic retinopathy), Alnylam (respiratory syncytial virus), and Merck (age-related macular degeneration, hepatitis C). Our random shRNA approach has potential applications in a variety of systems, including protection against viral infection (by retrieving shRNAs from surviving cells) and stem-cell differentiation (by flow-sorting cells with specific surface markers). Our study may serve as a proof of principle for these applications.

## Materials and Methods

### Library synthesis (Figure 1B)

All oligos from this section were made by IDT Inc., and all enzymes were obtained from New England Biolabs. We started by annealing a short primer (1) to a 97-nt oligo (2). The 97-nt oligo contains a 29-nt random sequence (N29) and multiple enzyme sites necessary for later steps. The oligo also contains five phosphorothioate linkages (indicated by the letter “s”) separating the last three A residues and the first three random residues. The 29-nt random sequence (N29) ends, in this case, with a G, so the library made from this particular oligo (N28+G) comprises one fourth of a complete, random N29 library. The primer (1) has two mismatches with respect to the oligo (2), designed to preserve the *Pme*I recognition sequence in the 97mer (2) and the *Aar*I recognition sequence in the primer (1). **Step 1**: A single extension was carried out with Klenow enzyme at 37°C for 30 min. **Step 2**: The extension product was ligated to a hairpin-loop linker (3) with T4 DNA ligase at 15°C overnight. **Step 3**: The ligated hairpin-loop was digested with Nb.*Bbv*CI, a nicking enzyme that cuts only one strand of DNA; this created an “exposed” area on the stem for annealing of the second primer (4). **Step 4**: The nicked hairpin-loop was “opened up” with the strand-displacing polymerase *Bst* at 65°C for 30 min, with a 10-fold molar excess of primer (4). **Step 5**: The product was digested with *Btg*ZI, which cuts downstream of its recognition site. The *Btg*ZI digestion was asymmetric because of the undigestible phosphorothioate linkages we designed. **Step 6**: The recessed end after *Btg*ZI digestion was filled in with Klenow to form a blunt end, and the other side was digested with *Not*I. **Step 7**: The DNA cassette was blunt-*Not*I ligated into an appropriately modified expression vector pSuper (Oligoengine). **Step 8**: *Pme*I and *Aar*I were used to digest the loop sequence; because of designed mismatches between primer (1) and the 97-nt oligo (2), *Pme*I cut on one side of the loop sequence and *Aar*I on the other. **Step 9**: The recessed end after *Aar*I digestion was filled in with Klenow to form a blunt end, and the vector was re-closed with a unimolecular, blunt-blunt ligation using T4 DNA ligase at 15°C overnight.

### Random Mutagenesis

Random mutagenesis of the 3p sequence was carried out on a PCR-MATE EP 391 DNA synthesizer (ABI biosystems) in our own laboratory. The manufacturer's instructions were followed, except that for each phosphoramidite bottle – A, G, C or T – small amounts of the other three phosphoramidites were spiked in. For example, if a 1% mutation rate were desired, then 1% of C, G, and T phosphoramidites would be mixed with 99% A. If C, G, and T incorporated into the oligo at equal efficiency, then 0.33% of each would be used. However, we adjusted for differences in incorporation efficiency (A = 20%, G = 26%, C = 24%, T = 30%). For our synthesis we aimed at 2 mutations per oligo, which comprised an 87-mer hairpin-loop structure plus flanking sequences. So the three “mutation” phosphoramidites were added for a combined total of 2.3% (2/87). The 87-mer sequences used to synthesize mutants of 3p were: 5′-GATCTCC-GTGGATCAGTGTGTTATAGCTCGGGCAGGCTAAACCCTGCCCGAGCTATAACACACTGATCCAC-TTTTTCAGGTCATCGC-3′; 5′- GGCCGCGATGACCTGAAAAA-GTGGATCAGTGTGTTATAGCTCGGGCAGGGTTTAGCCTGCCCGAGCTATAACACACTGATCCAC-GGA-3′. (Loop sequences are underlined, and the shRNA-encoding cassette is separated from the flanking sequences with a dash.) The synthesized oligos were purified using an oligo purification cartridge, per ABI instructions. Since the oligos have a strong propensity to form intramolecular hairpin-loop structures, we annealed the two oligos by mixing them at equal molar ratios and then renatured in a step-wise process using a PCR machine: 95°C×30 sec, 72°C×2 min, 37°C×2 min, and 25°C×2 min, followed by an incubation at 4°C. The annealed oligos were then phosphorylated with T4 kinase, and cloned into pSiren prepared with *Bgl*II and *Not*I and dephosphorylated with Shrimp Alkaline Phosphatase (Roche).

### Cell culture, retroviral transduction

The FL5.12 pro-B cell line was a gift from Dr. Craig Thompson (University of Pennsylvania). FL5.12 cells were cultured in RPMI 1640 media with 10% FBS (BioWhittaker), 10 mM Hepes pH 7.4, 100 U/ml Penicillin, 100 mg/ml Streptomycin, 55 mM β-Mercaptoethanol (all from Gibco), supplemented with 0.3 ng/ml IL3 (BD Pharmingen). To prepare retroviral supernatant for infection, 293T cells growing at ∼60% confluency were transfected with Effectene reagent (Qiagen) according to manufacturer's instructions. The pSiren library was co-transfected with an ecotropic retroviral packaging plasmid pCL-Eco (Imgenex) at a dose of 2.5 µg total DNA per well in a 6-well plate. Supernatant was harvested to infect FL5.12 cells with 3 cycles of centrifugation (1000 g×1 hr) and incubation (2 hrs), in the presence of 5 µg/ml polybrene (Sigma). Infection efficiency was monitored by GFP expression on a BD FACSCalibur flow cytometer. Ideally the GFP% was kept at ∼33% or less whenever a library was used to transduce cells, such that, by Poisson distribution, the majority of the infected cells received only one construct.

### Sequence retrieval and confirmation

To retrieve shRNA-encoding sequences integrated in the genome, cells that have been enriched for GFP after IL3 starvation/recovery cycles were pelleted, and their genomic DNA was extracted using a Qiagen kit. The shRNA-encoding cassette was amplified from genomic DNA using the following protocol: 95°C×5 min, 95°C/56°C/72°C at 30 s/45 s/10 min for 30 cycles, with primers flanking the shRNA-encoding cassette on pSiren. The sequences of the primers are 5′-CCGGAATTGAAGATCTGGG-3′ and 5′-CCGTAATTGATTACTATTAATAACTAGAATTC-3′. The long extension time (10 min) was necessary due to the hairpin structure of the template. To sequence the hairpin-loop structure we developed two sets of primers that amplify the two halves of the hairpin separately. To amplify the 5′ half of the stem, we used one primer upstream of the cassette and a second primer that comprises the loop sequence at its 3′ end and sequences downstream of the cassette at its 5′ end (thus “jumping over” the 3′ half of the stem). The sequences of these two primers are: upstream 5′-CCCCCTTGAACCCTTTATCC-3′ and downstream 5′-CGATGACCTGAAAAAGGTTTAGC-3′. The sequence of the 3′ half of the stem was obtained in a similar fashion; the primers were 5′-GGGCGTACTTGGCATATG-3′ and 5′-GACGGATCTCCGCTAAACC-3′. Conditions for these so-called “half-shRNA” PCR amplifications were: 95 C×5 min, 95°C/52°C/72°C at 30 s/45 s/5 min for 40 cycles.

### Apoptosis induction and caspase 3 assay

Apoptosis was induced in FL5.12 cells by washing 3× with IL3-negative medium and resuspending in IL3-negative medium. To replenish IL3 for recovery, IL3 was simply added back to the medium at 0.3 ng/ml. Tests of individual shRNAs were carried out with an overnight IL3 starvation of 14–22 hours. Cells were then stained with 20 µg/ml propidium iodide (PI, Sigma) in the presence of 0.2 mg/ml of RNAse A (Roche). The percentage of GFP-positive (infected), PI-negative (live) cells relative to the start of the experiment (just prior to the IL3 withdrawal) were determined by flow cytometry on a BD FACSCalibur. Caspase 3 was assayed using an enzyme activity kit (BIOMOL) according to manufacturer's instructions, by measuring the cleavage of a colorimetric substrate, Ac-DEVD-pNA.

### Statistical analysis

Pair-wise comparisons of means were conducted using Student's t-test. Error bars represent standard deviations. The data points for each bar graph were determined from 3 to 6 independent experiments.

## Supporting Information

Table S1 Enrichment for true-positive shRNA sequences after various rounds of selection and re-screening, assuming a true positive rate of only 1 in 1 million, false positive rates of either 1% (left) or 10% (right), and no heritability of false positives.
(0.03 MB DOC)

Figure S1 Sequencing data of the random library. (A) Sequencing results from the shRNA library before the creation of the non-complementary loop. At this stage, PmeI digest allowed the two halves of the stem to be separated and sequenced more easily. The electropherogram confirms all features of the cassette design. (B) Sequences of 40 random clones from the library. Note the last base is a G for all sequences, as discussed in the main text. In a perfectly random scenario, therefore, there should be 14 A/Ts and 15 G/Cs. The 40 clones showed a distribution of 14.78 A/Ts and 14.22 G/C, on average. Thus there is a very slight bias towards A/T. No obvious patterns in these sequences can be discerned.
(0.16 MB TIF)

Figure S2 Vector preparation for cloning of the shRNA-encoding cassette. (A) The cassette was initially designed for cloning into pSuper (Oligoengine); shown here are the steps used to prepare pSuper for blunt-end/NotI cloning. A linker containing the BbsI site was cloned into pSuper cut with BglII and MluI. The linker also contained the SnaBI site for the purpose of checking the ligation since there is another SnaBI site in pSuper. The altered pSuper was then digested with BbsI, which is a downstream cutter, and then filled-in with Klenow to form blunt ends on both sides. BbsI was positioned precisely on the linker, such that after the digest and fill-in, the blunt end is at the correct distance from the promoter per the manufacturer's instructions. The blunt-blunt vector was then cut with NotI to form the blunt-end/NotI pSuper, ready for ligation of the shRNA-encoding cassette (Figure 1B) (B) Transfer of the library en bloc into pSiren (Clontech) to ensure consistent GFP expression after retroviral infection and integration. The standard restriction sites for cloning into pSiren are BamHI and EcoRI. We introduced a NotI site between the BamHI and EcoRI sites using a primer with all three sites in the correct order. This primer also mismatches the pSiren template by “jumping over” two bases highlighted in the figure. These two bases needed to be eliminated because the cassette excised from pSuper with BglII-NotI had two extra bases, “CC,” after the BglII site (see part (A)). Therefore, paired with an appropriate upstream primer, we introduced a NotI site and eliminated two bases in pSiren. The modified vector was then cut with BamHI and NotI, and was ready to accept the BglII-NotI cassette from pSuper, with the correct spacing from the PolIII promoter. Note that BamHI and BglII have compatible cohesive ends. The two primer sequences are: “RINotBam” 5′-CTTGAATTCGCGGCCGCTTGGATCCGTCCTTTCCACAAG-3′, and “SirenBgl” 5′-CCGGAATTGAAGATCTGGG-3′.
(0.08 MB TIF)

Figure S3 8p offers delayed protection of FL5.12 cells from IL3 withdrawal. Initially, 8p did not offer protection (Figure 3), but its effect became apparent one week later. Survival percentages were obtained in the same manner as for all other survival figures, after IL3 starvation of 22 hours in this experiment. (p = 0.019 for 1p vs. ctrl, p<0.01 for 3p or 8p vs ctrl) Data are means +/− SD.
(0.04 MB TIF)

Figure S4 1p, 3p and 8p do not offer FL5.12 cells a growth advantage. Cells were infected with 1p, 3p, 8p, or pSiren and compared with cells infected with the same random control shRNA (ctrl). GFP% was 20–25% after the infections. Cells were then seeded at 500,000/2 ml (relative cell number  = 1) in a 12-well plate and allowed to grow for three days. None of the three hit shRNAs offered any growth advantage over the random control shRNA. The growth rate was also comparable to the GFP-negative cells in the same culture. Data are means +/− SD.
(0.06 MB TIF)

Figure S5 Effects of the hit shRNAs are long lasting. Cells were cultured in IL3+ media continuously for 4 months. IL3 was then withdrawn for 20 hours, and protective effects were still seen (p = 0.0087 for 1p vs. ctrl, p = 0.073 for 3p vs. ctrl, p = 0.012 for 8p vs. ctrl). Data are means +/− SD.
(0.02 MB TIF)

Figure S6 10 random clones offered no protection. FL5.12 cells were transduced with 10 additional random clones from the 300K library, along with pSiren, or the 300K library itself. IL3 was withdrawn for 15 hours, and the survival percentage was similar in all cases. A positive control was not included in this experiment; however, we have performed such starvation assays more than 50 times under different conditions, and the survival percentages shown in this figure are seen consistently. Data are means +/− SD.
(0.03 MB TIF)
