# Supplementary material for: A Random shRNA-Encoding Library for Phenotypic Selection and Hit-Optimization
Source: PLoS One. 2008 Sep 9;3(9):e3171. doi: 10.1371/journal.pone.0003171 (PMC2525836; doi:10.1371/journal.pone.0003171)
Supplement: Table S1 — Enrichment for true-positive shRNA sequences after various rounds of selection and re-screening, assuming a true positive rate of only 1 in 1 million, false positive rates of either 1% (left) or 10% (right), and no heritability of false positives. (0.03 MB DOC) [file pone.0003171.s001.doc]

**Table S1.** Enrichment for true-positive shRNA sequences after various rounds of selection and re-screening, assuming a true positive rate of only 1 in 1 million, false positive rates of either 1% (left) or 10% (right), and no heritability of false positives.

|  | True Positives | False Positives (1%) |  | True Positives | False Positives (10%) |
| --- | --- | --- | --- | --- | --- |
| Round 1 | 1 | 10,000 |  | 1 | 100,000 |
| Round 2 | 100 | 10,000 |  | 10 | 100,000 |
| Round 3 | ~10,000 | 10,000 |  | 100 | 100,000 |
| Round 4 | ~500,000 |  |  | 1000 | 100,000 |
| Round 5 |  |  |  | ~10,000 | 100,000 |
| Round 6 |  |  |  | ~100,000 | 100,000 |
| Round 7 |  |  |  | ~500,000 |  |
