# Supplementary figures and images for: A Random shRNA-Encoding Library for Phenotypic Selection and Hit-Optimization
Source: PLoS One. 2008 Sep 9;3(9):e3171. doi: 10.1371/journal.pone.0003171 (PMC2525836; doi:10.1371/journal.pone.0003171)

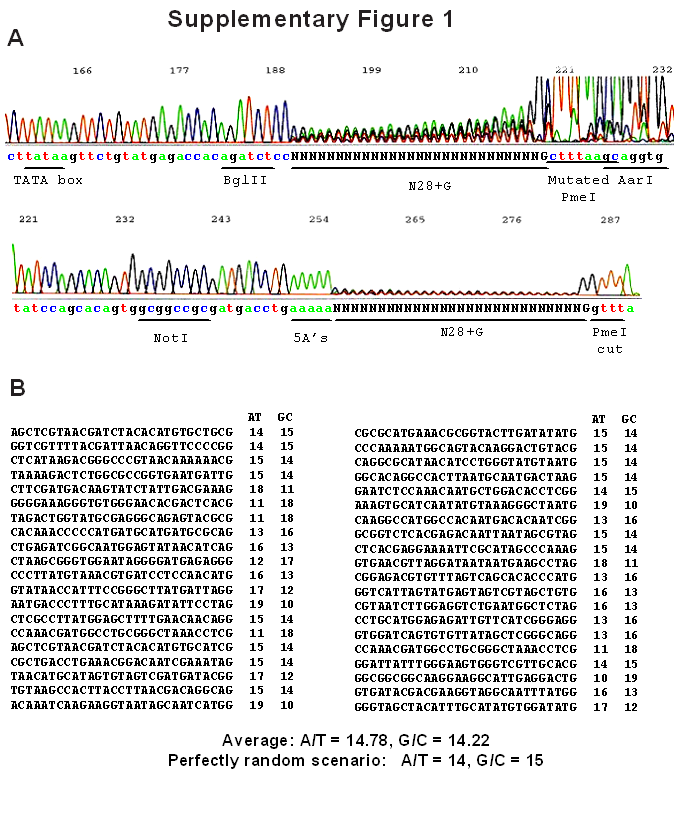

Supplement: Figure S1 — Sequencing data of the random library. (A) Sequencing results from the shRNA library before the creation of the non-complementary loop. At this stage, PmeI digest allowed the two halves of the stem to be separated and sequenced more easily. The electropherogram confirms all features of the cassette design. (B) Sequences of 40 random clones from the library. Note the last base is a G for all sequences, as discussed in the main text. In a perfectly random scenario, therefore, there should be 14 A/Ts and 15 G/Cs. The 40 clones showed a distribution of 14.78 A/Ts and 14.22 G/C, on average. Thus there is a very slight bias towards A/T. No obvious patterns in these sequences can be discerned. (0.16 MB TIF) [file pone.0003171.s002.tif]

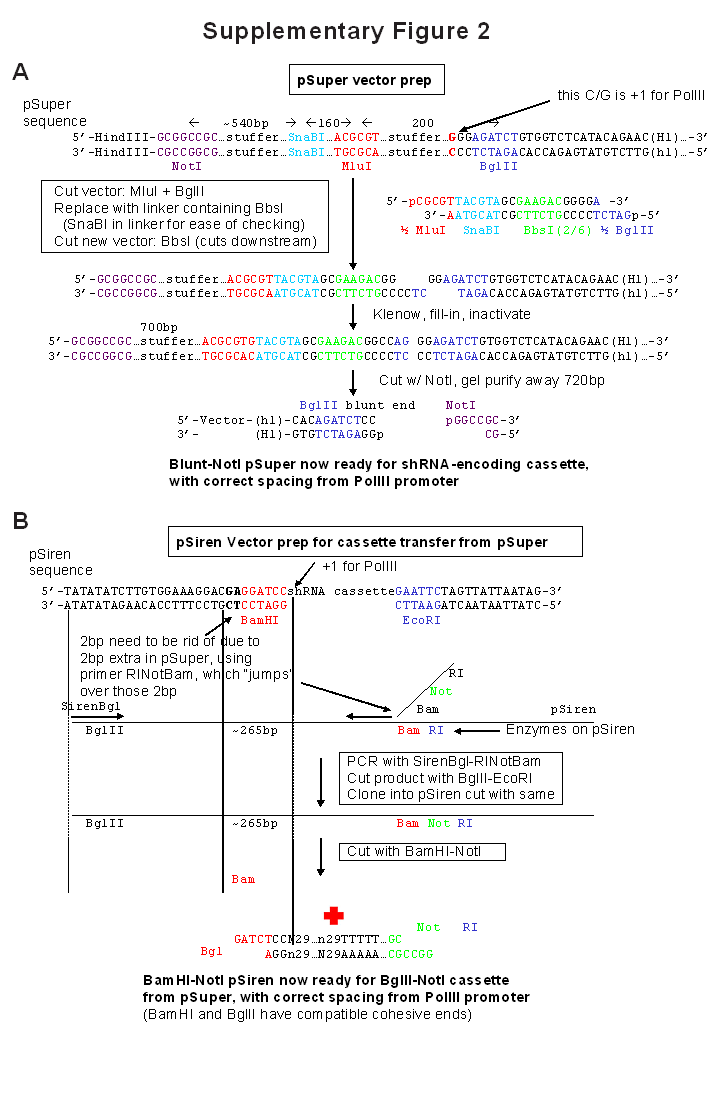

Supplement: Figure S2 — Vector preparation for cloning of the shRNA-encoding cassette. (A) The cassette was initially designed for cloning into pSuper (Oligoengine); shown here are the steps used to prepare pSuper for blunt-end/NotI cloning. A linker containing the BbsI site was cloned into pSuper cut with BglII and MluI. The linker also contained the SnaBI site for the purpose of checking the ligation since there is another SnaBI site in pSuper. The altered pSuper was then digested with BbsI, which is a downstream cutter, and then filled-in with Klenow to form blunt ends on both sides. BbsI was positioned precisely on the linker, such that after the digest and fill-in, the blunt end is at the correct distance from the promoter per the manufacturer's instructions. The blunt-blunt vector was then cut with NotI to form the blunt-end/NotI pSuper, ready for ligation of the shRNA-encoding cassette (Figure 1B) (B) Transfer of the library en bloc into pSiren (Clontech) to ensure consistent GFP expression after retroviral infection and integration. The standard restriction sites for cloning into pSiren are BamHI and EcoRI. We introduced a NotI site between the BamHI and EcoRI sites using a primer with all three sites in the correct order. This primer also mismatches the pSiren template by “jumping over” two bases highlighted in the figure. These two bases needed to be eliminated because the cassette excised from pSuper with BglII-NotI had two extra bases, “CC,” after the BglII site (see part (A)). Therefore, paired with an appropriate upstream primer, we introduced a NotI site and eliminated two bases in pSiren. The modified vector was then cut with BamHI and NotI, and was ready to accept the BglII-NotI cassette from pSuper, with the correct spacing from the PolIII promoter. Note that BamHI and BglII have compatible cohesive ends. The two primer sequences are: “RINotBam” 5′-CTTGAATTCGCGGCCGCTTGGATCCGTCCTTTCCACAAG-3′, and “SirenBgl” 5′-CCGGAATTGAAGATCTGGG-3′. (0.08 MB TIF) [file pone.0003171.s003.tif]

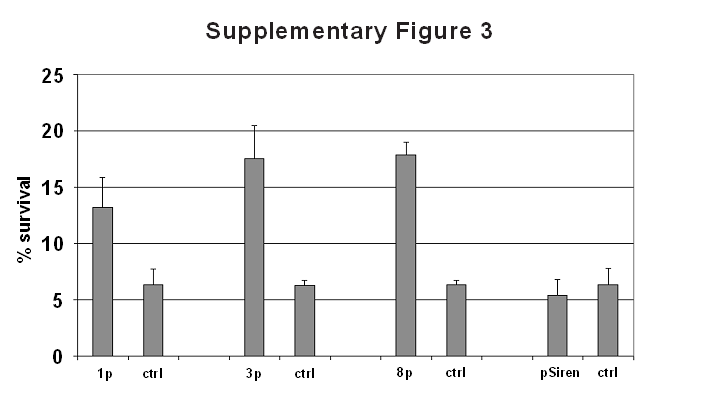

Supplement: Figure S3 — 8p offers delayed protection of FL5.12 cells from IL3 withdrawal. Initially, 8p did not offer protection (Figure 3), but its effect became apparent one week later. Survival percentages were obtained in the same manner as for all other survival figures, after IL3 starvation of 22 hours in this experiment. (p = 0.019 for 1p vs. ctrl, p<0.01 for 3p or 8p vs ctrl) Data are means +/− SD. (0.04 MB TIF) [file pone.0003171.s004.tif]

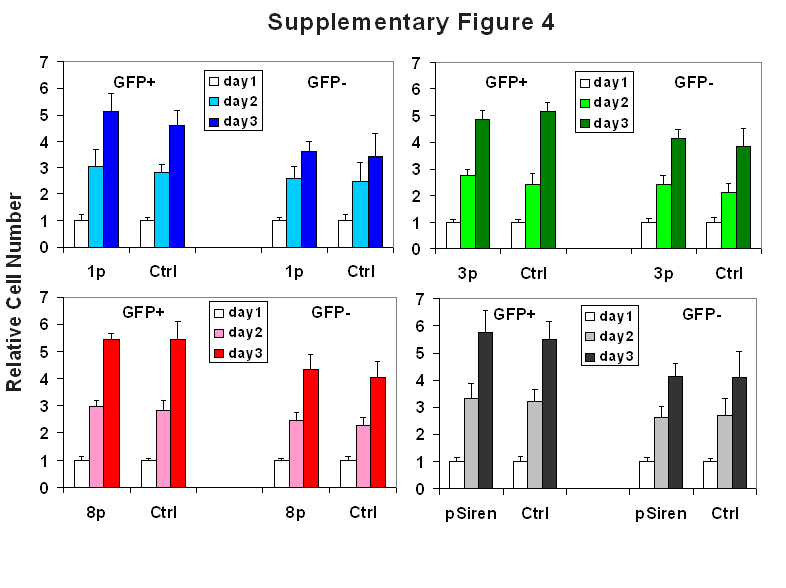

Supplement: Figure S4 — 1p, 3p and 8p do not offer FL5.12 cells a growth advantage. Cells were infected with 1p, 3p, 8p, or pSiren and compared with cells infected with the same random control shRNA (ctrl). GFP% was 20–25% after the infections. Cells were then seeded at 500,000/2 ml (relative cell number = 1) in a 12-well plate and allowed to grow for three days. None of the three hit shRNAs offered any growth advantage over the random control shRNA. The growth rate was also comparable to the GFP-negative cells in the same culture. Data are means +/− SD. (0.06 MB TIF) [file pone.0003171.s005.tif]

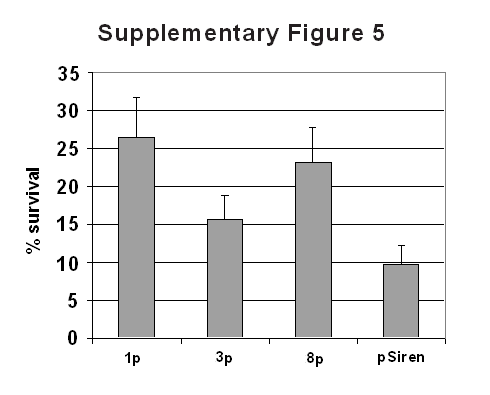

Supplement: Figure S5 — Effects of the hit shRNAs are long lasting. Cells were cultured in IL3+ media continuously for 4 months. IL3 was then withdrawn for 20 hours, and protective effects were still seen (p = 0.0087 for 1p vs. ctrl, p = 0.073 for 3p vs. ctrl, p = 0.012 for 8p vs. ctrl). Data are means +/− SD. (0.02 MB TIF) [file pone.0003171.s006.tif]

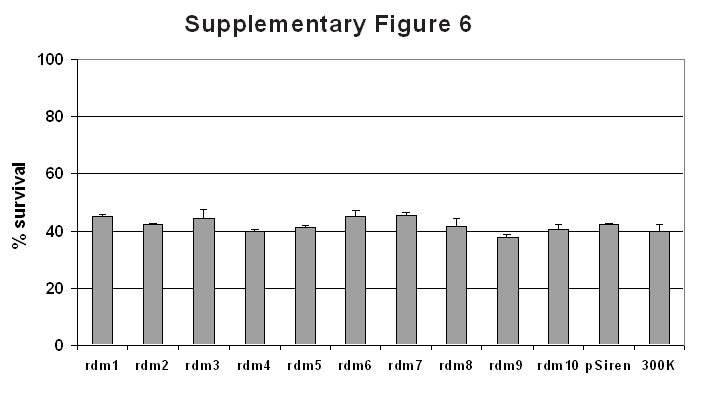

Supplement: Figure S6 — 10 random clones offered no protection. FL5.12 cells were transduced with 10 additional random clones from the 300K library, along with pSiren, or the 300K library itself. IL3 was withdrawn for 15 hours, and the survival percentage was similar in all cases. A positive control was not included in this experiment; however, we have performed such starvation assays more than 50 times under different conditions, and the survival percentages shown in this figure are seen consistently. Data are means +/− SD. (0.03 MB TIF) [file pone.0003171.s007.tif]
